# Natural Compounds and Breast Cancer: Chemo-Preventive and Therapeutic Capabilities of Chlorogenic Acid and Cinnamaldehyde

**DOI:** 10.3390/ph17030361

**Published:** 2024-03-11

**Authors:** Yusuff Olayiwola, Lauren Gollahon

**Affiliations:** 1 Department of Biological Sciences, Texas Tech University, 2500 Broadway, Lubbock, TX 79409, USA; yolayiwo@ttu.edu; 2 Obesity Research Institute, Texas Tech University, 2500 Broadway, Lubbock, TX 79409, USA

**Keywords:** natural compounds, phytochemicals, chlorogenic acid, cinnamaldehyde, breast cancer

## Abstract

Globally, breast cancer is not only the most frequently diagnosed cancer but also the leading cause of cancer death in women. Depending on breast cancer histotype, conventional breast cancer treatment options vary greatly in efficacy and accompanying side effects. Thus, there is a need for more effective and safer strategies that impact breast cancer at all stages. Plant-based natural products are easily available, with them proving effective and inexpensive. Two such phytochemicals are chlorogenic acid and cinnamaldehyde. Studies have shown their efficacy against different molecular subtypes of breast cancers in vitro and in vivo. In this review, we discuss their current status in anticancer research with specific emphasis on chlorogenic acid and cinnamaldehyde. We describe their multiple mechanisms of action in destroying breast cancer cells, their potential uses, and the need for translational applications. We also include future directions for investigations to progress chlorogenic acid and cinnamaldehyde research from bench to bedside.

## 1. Introduction

The morbidity and mortality of cancer remain high in our society despite intensive biomedical research in oncological science. The incidence of breast cancer continues to rise globally, along with the mortality rate. Traditional breast cancer treatment options are not sustainable. Therefore, the need for effective and safer strategies arises [1]. However, discovering effective cancer treatments has been viewed as a quest for the “Holy Grail”. This is primarily because various cancer treatment approaches are ineffective, and even pose toxicological impacts to cancer patients. Cancer stem cell subpopulations, multi-mechanistic drug resistance development, epigenetic reprogramming, off-target impacts or non-specificity of cancer treatments, and cancer cell metastasis are the major factors constituting the bane of effectively addressing the menace of neoplastic cell transformation [2].

Different innovative methods are constantly being evaluated for the development of safe and effective anticancer strategies. These approaches include the use of targeted therapy and immunotherapy, gene therapy, tumor ablation, magnetic hyperthermia, and phytochemicals among others [3]. Of all these, the use of natural products, either as whole extracts or combinations of their phytochemical parts, has gained more attention for their medicinal qualities. Bioactive phytochemical agents present in fruits, vegetables, spices, legumes, and grains have been described to be promising in cancer treatment approaches. They are inexpensive, readily available, safe, and effective in the treatment of a variety of diseases [4]. Additionally, the evolutionary history of humans using plants as medicinal and nutrient sources provides an abundant source of naturally occurring products that have been shown to kill abnormal cells while sparing normal cells—thus preventing off-target toxicity associated with conventional therapies [4,5]. Well-known examples of plant-based natural products, which are also called phytochemicals, are curcumin, carotenoids, genistein, daidzein, resveratrol, ellagic acid, punicalagin, and silibinin [4]. Phytochemical agents have displayed anti-inflammatory, anti-proliferative, anti-metastatic, antiviral, pro-apoptotic, and free radical scavenging capacities in vitro and in vivo [6,7,8,9]. Moreover, research into natural compounds as an alternative method in cancer treatment has received overwhelming interest owing to their relative safety and ability to affect multiple molecular targets in cancer cells. Phytochemical agents have been reported to show effectiveness against different types of cancer including various types of breast cancer through varied mechanistic processes such as the induction of apoptosis, the inhibition of DNA replication, the inhibition of telomerase, the dysregulation of mitotic cell division, angiogenic and metastatic process inhibition, and the induction of differentiation in cancer cells [6,7,8,9,10,11].

Prior studies, as well as work from our lab, have demonstrated chlorogenic acid (CGA) and cinnamaldehyde (CA) as two important plant-based bioactive agents with a strong ability to kill different types of breast cancer cells both alone and synergistically with other phytochemical agents by modifying the functions and structures of various cellular proteins and thus several pathways such as PI3K/Akt, MAPK, Nrf2, Wnt/β-catenin, p53 and NF-κB to cause cytotoxicity to breast cancer cells [11,12,13,14,15,16,17,18,19,20,21]. Thus, the purpose of this review is to more deeply explore studies that report the capability of chlorogenic acid and cinnamaldehyde in effectively killing various molecular subtypes of breast cancer and put this information into a more global perspective. Furthermore, we discuss future directions for investigating the anticancer capacity of the small molecules’ mixture in preclinical studies considering recent NIH interest in natural products’ extracts as potential anticancer treatments.

## 2. Conventional Treatment Methods for Breast Cancer and Their Challenges

Cancer is a complex and dynamic disease condition that is often characterized by heterogeneous cancer cells as the disease progresses [22]. Cancer heterogeneity is defined as different cancer cells exhibiting various morphological and phenotypic characteristics, such as differential gene expression patterns, cellular morphology, metabolism, proliferation, and metastatic capability. This characteristic, which could be inter-tumoral and intra-tumoral, is responsible for diverse populations of cancer cells observable in a given tumor [22,23]. One of the major challenges facing breast cancer treatment is the heterogeneity of the disease. Consequent to this heterogeneity, the majority of breast tumors may be made up of a diverse group of cells with various levels of sensitivity to treatment and distinctive molecular signatures [22,23,24,25]. It becomes important to characterize each tumor subtype to know the appropriate approaches to employ for treatment. Thus, the treatment of breast cancer is dependent on the molecular subtype and phenotypic characterization of the tumor, as well as the disease stage and tumor grade. A detailed review of these molecular subtypes can be found in other literature [26,27,28]. The molecular subtypes of breast cancer are strong prognostic and predictive factors [29].

The various conventional methods for treating breast cancer can be classified as local or systemic depending on whether the treatment is delivered explicitly on the mass of tumor cells without affecting all other parts of the body or delivered into the systemic circulation to reach and affect all parts of the body where the tumor cells may reside. Local treatments include surgery, which could be mastectomy or lumpectomy, and radiotherapy. Systemic treatment can be administered orally or muscularly or delivered into the blood circulation. They include chemotherapy, hormone therapy, immunotherapy, and targeted therapy [25,30,31].

Although different treatment approaches used for treating different types of breast cancers can kill cancer cells to some extent, they are often associated with varied complications. There are concerns about the safety, toxicity, and effectiveness of the methods. Chemotherapy is aimed at inhibiting tumor growth and proliferation by causing cell cycle arrest at various phases of the cell cycle of actively dividing cells. They primarily affect cell macromolecular synthesis and function by interfering with DNA, RNA, or protein synthesis [32]. However, chemotherapy cocktails are not able to distinguish between actively proliferating cancer cells and normal cells such as cells of skin cells, cells in the bone marrow that generate different types of blood cells, and cells in the gastrointestinal tract, mouth, and reproductive tissues [32,33]. Most of the cancer drug agents are metabolized and excreted by either the liver and/or kidney. As a result, the organs are exposed to the toxicity of bioactivated drugs. Toxic metabolites of the drugs can accumulate in the organs, leading to organ dysfunction or even mutational events and carcinogenesis [32].

Chemotherapy can result in a weakened immune system, anemia, alopecia, and other conditions such as fatigue, nausea, diarrhea, and mouth sores, among others [32,33,34]. Surgery does not always result in total removal of the tumor as remnants of breast cancer cells can grow and multiply, resulting in remission. Similarly, vital tissues can be affected during the surgical removal of the tumor. Radiation therapy can result in bystander effects in healthy cells, thereby causing DNA fragmentation and mutation in the cells, which can lead to novel neoplastic conditions. The unintended consequences of damaging normal cells and the destruction of vital tissue in the proximity of the targeted tumor associated with cancer radiotherapy limit its efficacy. Immunotherapy mainly causes autoimmune diseases as a result of overactivation of the immune cells. Targeted therapy is equally associated with several complications. Because the drugs are delivered into the systemic circulation, they target normal cells that bear resemblance to the cancer cells they are designed to target [35]. Some of these side effects are related to the impact of the target and may serve as surrogates for an anti-tumor response. Other toxicities may be caused by the type of agent used, such as antibodies versus small molecular inhibitors, or effects on targets unrelated to tumor response. Dyslipidemia/hyperglycemia, ocular toxicity, skin rash, hypertension, hypothyroidism, and proteinuria are among the side effects of targeted therapy [35,36].

## 3. The Need for Safer/More Effective Alternative Breast Cancer Treatment Approaches

Among the obstacles to breast cancer treatment options, drug resistance and ineffectiveness and lack of safe drug delivery systems are the biggest problems in cancer therapies [37]. The efficiency of traditional cancer therapies is limited due to tumor pathology, the architectural abnormality of the tumor tissue vascular system, and inter- and inter-tumoral differences as well as the inherent toxicity of the therapy [22,37,38]. Since the broad aim of medical science is to prevent disease development in healthy humans and cure or treat diseased individuals, it therefore becomes imperative to find holistic solutions to breast cancer [39]. Holistic breast cancer treatment has been described as the “Holy Grail” especially in light of the current breast cancer treatment options being depicted as “Achilles heels” as cancer cells have been able to develop resistance to circumvent the cytotoxic agents. Ideally, cancer cells with all their vulnerabilities are supposed to be the figurative “Achilles heels”. But the reality is the opposite. Current treatment modalities are not achieving their objective of effectively and safely killing cancer cells and preventing their recurrence. However, because we must address the menace of breast cancer that is projected to kill more than a million women and produce over three million morbidities annually by 2040 [40], we must search for the metaphorical “Holy Grail” that will not only optimally kill cancer cells but also prevent cancer relapse, as well as sparing normal cells from its cytotoxicity [41].

While other methods have shown simultaneous promise and challenges, the use of plant-derived natural compounds has been proven to hold the much-sought-after Ehrlich’s “poison arrow”. This is because of their unmatched effectiveness in their cytotoxic effects against breast cancer cells and other types of neoplastic diseases, their bioavailability, and safety to normal cells as well as their capability to prevent cancer relapse [4,5,7,8,9,10,42,43,44].

## 4. Breast Cancer and Chemoprevention Characteristics of Natural Compounds

Cancer chemoprevention is a pharmacological modality using natural or synthetic chemical agents to prevent, suppress, or delay the initiation of carcinogenesis or to inhibit premalignant cells from becoming malignant and invasive diseases [45,46,47,48]. Mechanistically, different chemo-preventive agents elicit their chemoprevention by affecting various molecular steps in different stages of carcinogenesis (initiation, promotion, and development). They impact multiple molecular targets to prevent, delay, or reverse tumor initiation, promotion, and/or development. They can be classified as either blocking or suppressing agents, with blocking agents defined as targeting cancer initiation events while suppressing agents act to prevent tumor promotion [45,46].

One of the mechanisms through which blocking agents act is the induction of xenobiotic metabolizing enzymes. Phase I metabolism includes such reactions as oxidation, reduction, and hydrolysis and serves to increase the hydrophilicity of non-polar compounds via the introduction of functional groups like -NH_2_ or -OH_2_ through the enzymatic activities of various isoforms of cytochrome P450 (CYPs). Although phase I biotransformation reactions could result in the bioactivation of potent carcinogens, some reactions can deactivate or detoxify active carcinogens and mutagens and subject them to removal from systemic circulation. Phase II drug metabolic reactions are the conjugation (such as glucuronidation, acetylation, methylation, and glutathionylation) of molecular moieties to the products of phase I reactions (or the xenobiotics that do not experience phase I biotransformation reactions) to make them more polar and aid their excretion from the body system. Enhancement of the detoxification of mutagens, carcinogens, and reactive oxygen/nitrogen species (ROS/RNS) by the enzymes of the xenobiotic metabolic pathway has been established as a method through which phytochemicals can elicit cytoprotective effects in their chemoprevention to prevent the initiation of carcinogenesis. Some phytochemicals are monofunctional, in that they can induce the expression and activities of phase II enzymes alone, or bifunctional, such that they serve as inducers of both phase I and phase II enzymes. Studies have shown polyphenolics, such as flavonoids, stilbenes, lignans, tannins, curcumin, and coumarins, and sulfur-containing chemicals (glucosinolates), such as isothiocyanates, to be blocking agents that induce the expression of detoxification enzymes [46,49].

By inducing the expression of detoxification enzymes, chemo-preventive agents prevent events like mutation, DNA damage, and genetic instability that can initiate neoplastic cell transformation from taking place. The induction of phase II detoxifying enzymes has been elucidated to proceed through activation of the Nrf2-ARE pathway [45,46,49,50].

Suppressing agents, on the other hand, inhibit the tumor promotion and progression in already transformed cells and promote their removal from the tissue mass. Phytochemical agents eliciting suppressive effects in their chemoprevention include isothiocyanates, which are found in broccoli, watercress, cauliflower, and cabbage; flavonoids found in parsley, blueberries, grapefruits, and citrus fruit; and coumarins found in cinnamon, sweet clover, and tonka beans [45,49]. Suppressive agents inhibit tumor promotion through several mechanisms which include the inhibition of apoptosis, cell cycle arrest, the inhibition of angiogenesis and pathways sustaining cancer cells such as nuclear factor kappa B (NFκB), and the induction of cell differentiation [45,49].

The induction of apoptosis by phytochemicals proceeds in a mitochondrial-dependent manner by inhibiting the release of anti-apoptotic proteins such as Bcl-2 and Bcl-xL and promoting the release of pro-apoptotic proteins such as Bak and Bax, which results in the release of cytochrome C from the inner mitochondrial membrane and the formation of an apoptosome. Once the apoptosome is formed, effector proteases, caspases 3, 6, and 7, are activated and released. This results in the degradation of intracellular proteins, cell shrinkage, nuclear fragmentation, and blebbing. Similarly, suppressing agents can block hormone synthesis and hormone receptors in breast cancer cells that depend on hormones for promotion and development [45,46,49,51,52].

## 5. Therapeutic Potential of Natural Compounds in Breast Cancer

**Figure 1:**
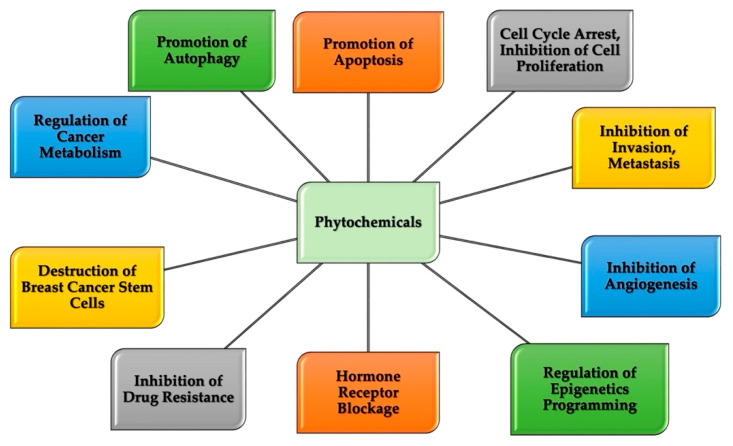
Mechanisms of actions of phytochemicals in breast cancer cells: Phytochemicals elicit their antineoplastic potential through several mechanisms indicated here. Various pathways that are central to the progression and aggressiveness of cancer cells are often impacted by phytochemicals, including multi-drug resistance and metastasis.

As described previously, the current modalities for breast cancer treatment are not always effective or safe and are overly expensive. There is a need to find approaches that will overcome these mitigating factors. Phytochemicals appear to hold the key to an effective treatment strategy. Various bioactive agents in plants have demonstrated, both in vivo and in vitro, the ability to target different breast cancer molecular subtypes and breast cancer stem cell populations. Figure 1 illustrates the identified mechanisms of actions of phytochemicals in breast cancer cells. Phytochemicals are effective, non-toxic, and widely available and have several biological activities including anti-inflammatory, anti-angiogenic, anti-proliferative, antioxidant, pro-apoptotic, and anticancer properties [4,5,53,54].

The luminal subtype of breast cancer, having both estrogen and progesterone receptors (ER/PR), is treated with hormonal therapy, which works by either blocking hormone synthetic pathways, disrupting the availability of natural hormones, or blocking cell surface hormone receptors [36]. However, breast cancer cells develop resistance to hormone–therapeutic agents. Phytochemicals have demonstrated effectiveness in acting as estrogen and progesterone hormone antagonists by blocking the receptor binding sites. They act as selective estrogen receptor modulators (SERMs) by making the ER binding site unavailable for their hormonal ligands, thereby shutting down the hormone-mediated breast cancer cell proliferation and decreasing overall survival. By this mechanism, phytochemicals could be considered cytostatic to breast cancer cells because they slow down cell cycle processes via downstream intracellular processes [36,55]. Such phytochemicals include isoflavones obtained from legumes; lignans found in a wide variety of seeds, whole grains, and vegetables; and coumestans present in pinto and lima beans. Genistein, a natural flavone compound found in soy, exerts significant antiproliferative insults on ER+ human breast carcinoma cells through the induction of p21 expression, G2-M arrest, and apoptosis [56]. Epigallocatechin gallate (EGCG), a major catechin found in green tea, shut down the angiogenic and proliferative capacity of breast cancer cells through the inhibition of hypoxia-inducible factor 1 subunit alpha (HIF-1α), NFκB activation, and vascular endothelial growth factor (VEGF) expression in a murine model [11,57]. Sen and Chatterjee (2011) [58] showed that EGCG downregulates EGF-induced matrix metalloproteinase-9 (MMP9) in ER+ breast cancer cells, thereby shutting down cell invasion and metastasis [58]. An in vivo and in vitro investigation established that quercetin, a plant flavonol obtained from red onions, capers, and kale, causes apoptotic cell death in both MCF7 and CAL51 cell lines, indicating the ability of the compound to target both estrogen-receptor positive and triple-negative breast cancer cell lines [59].

The basal-like breast cancer subtype, commonly known as triple-negative breast cancer (TNBC), is the most aggressive subtype of breast cancer, characterized by remission, and it defiles several treatment options as they lack all of the three types of hormone receptors that are often targeted for breast cancer treatments. Curcumin, resveratrol, 6-gingerol, capsaicin, black currant extract, and EGCG were established to potently inhibit the migration of metastatic triple-negative breast cancer cells [53,60]. According to Wu et al. (2020) [61], capsaicin, a bioactive agent found in chili pepper, inhibits the overall survivability of breast cancer cells through mediation of the complex CDK8/PI3K/Akt/Wnt/β catenin signaling pathway [61]. An in vitro and in vivo mouse model evaluation of chalcones, polyphenolic compounds in the flavonoid family, demonstrates their anticancer capacity against TNBCs while sparing normal cells [62]. The growth and metastasis of MDA-MB-231 cells, a highly aggressive and metastatic TNBC cell line, was reported to be inhibited via modulation of PI3K/Akt and NFκB pathways by myriads of phytochemicals in blueberry according to Adams and colleagues (2010) [63]. Phytochemicals employ different mechanistic processes to downregulate pathways that promote the aggressiveness of TNBCs, as further elucidated by the findings of Li and colleagues (2012) [64] in their investigation of the anticancer roles of ganoderic acids. Ganoderic acids are triterpenoids found in mushrooms. They inhibit the growth, migration, and invasiveness of TNBCs by suppressing the activities of transcription factors NFκB and AP-1, resulting in suppression of the secretion of uPA and the downregulation of Cdk4 expression [64]. Ajwa date fruits, which have been part of the essential diet of the Arabs and their neighbors since time immemorial, have been investigated for their phytochemical constituent and pharmacological importance. They contain such bioactive agents as phenolics, flavonoids, and terpenoids. Khan et al. (2021) [65] showed that extract from Ajwa dates pulp promotes apoptotic cell death in human triple-negative breast cancer through modulation of Bcl-2 family proteins and inhibition of the AKT/mTOR pathway [65].

Breast cancer stem cells (BCSCs) are a small population of cells present in breast tumors capable of self-renewal, initiating, and differentiating into different molecular subtypes of breast cancer cells, and thus contributing to breast cancer heterogeneity. They are largely responsible for tumor metastasis, drug resistance, and tumor relapse. Breast cancer stem cells are sustained by several signaling pathways such as Wnt, Hippo, Notch, and Hedgehog signaling pathways as well as JAK/STAT, PI3K/AktmTOR, and Wnt/β-catenin pathways [66,67,68]. Targeting BCSCs is imperative in improving the efficacy of breast cancer treatment. Current breast cancer treatments are not able to destroy the BCSC population, thus the occurrence of breast cancer remission [69]. Phytochemicals, owing to their ability to affect multiple pathways and molecular targets, have proved effective in destroying the BCSC population and improving treatment responses in breast cancer therapeutic research.

Curcumin, the principal curcuminoid of turmeric associated with myriads of medicinal potentials, was revealed by Mukherjee et al. [70] to inhibit the migratory capability of BCSCs through the amplification of the E-cadherin/β-catenin negative feedback loop. Nuclear translocation of β-catenin in BCSCs has been mechanistically linked with the downregulation of E-cadherin expression, reduced E-cadherin/β-catenin complex formation, and upregulation of slug and snail pathways. These together result in the upregulation of epithelial–mesenchymal transition (EMT) in the epithelial cells and thus the migratory and invasive capability of the BCSCs. Curcumin shuts down the canonical wnt/β-catenin pathway, thereby preventing nuclear translocation of β-catenin and activation of the Slug transcription factor. This results in the restoration of E-cadherin expression and inhibition of EMT and BCSC migration [70]. Furthermore, curcumin shuts down breast cancer stemness-maintaining pathways, promotes apoptosis, and inhibits the proliferative potential of BCSCs. Sonic hedgehog and Wnt/β-catenin pathways are the key signaling pathways in cancer stem cells and are responsible for aggressiveness, heterogeneity, drug resistance, and remission in breast cancer. However, Li et al. [71] reported that curcumin was able to shut down the pathways of MCF7 and SUM159 sphere-forming cells. The study revealed that the phytochemical kills BCSCs because of the downregulation of the two pathways [71]. Resveratrol, a polyphenolic compound found in cranberries, peanuts, and blueberries, elicits the ability to downregulate the Wnt/β-Catenin signaling pathway and promote autophagy in breast cancer stem cells [72]. Dioscin, a widely distributed saponins found in plants, are known in ethnopharmacology science for its anti-inflammatory, antioxidative, antitumor, and immunostimulatory roles. Ock and Kim (2021) [73] investigated its impact on breast cancer stemness. It was found that the bioactive agent decreases the breast cancer stemness properties through cell cycle arrest by regulating p38 MAPK and AKT/mTOR signaling pathways. In an in vitro study, the natural compound promotes the expression of p21 and p53 and inhibits the expression of various cyclins and cyclin-dependent kinases (CDKs). The compound prevents the proliferation of BCSCs as a result of cell cycle arrest and modulation of p38 MAPK and AKT/mTOR pathways [73].

Quercetin is an important flavonoid that belongs to the polyphenol class of plant secondary metabolite and found in various fruits and vegetables with varied pharmacological potentials [59,74]. Wang and his group (2018) [74] reported that the phytochemical can modulate breast cancer stemness properties through myriads of mechanisms [74].

The phytochemical downregulates the activity of aldehyde dehydrogenase 1A1 (ALDH1A1), an enzyme whose high expression level corresponds to the increased clonogenicity, tumorigenicity, invasiveness, and stemness properties of breast cancer. Quercetin also suppresses the expression of Mucin 1 (MUC1), a transmembrane glycoprotein overexpressed in breast cancer cells because of gene amplification and the loss of gene transcription and post-transcription regulatory networks. MUC1 interacts with EGFRs to activate cell proliferation-related signaling cascades, and it has been implicated in the invasion and metastasis of different types of tumors including breast cancer. Thus, inhibition of MUC1 by the bioactive agent inhibits breast cancer cell proliferation and metastasis [74]. Similarly, the natural product downregulates epithelial cell adhesion molecule (EpCAM) expression, a protein at the center of cancer therapeutic research which is widely reported to play important roles in cancer stemness, cell proliferation, metabolism, and angiogenesis, EMT, and drug resistance in breast cancer cells and other types of carcinomas. EpCAM undergoes crosstalk with several bio-signaling networks that are important for cancer stem cell maintenance and cancer survival. It is reported to crosstalk with Wnt/β-catenin, PI3K/AKT/mTOR, TGF-β/SMAD, and p53 pathways to maintain carcinoma stemness and the overall survival of epithelial tumors [75]. Put together, the findings of Wang and colleagues (2018) [74] indicate the impact of quercetin, an important polyphenol present in fruits and vegetables, in shutting down the stemness characteristic of breast cancer progenitor cells [74].

Binienda et al. [76] reported the role of silibinin, the main active component in silymarin, in affecting different molecular targets in breast cancer cells. The compound influences the activity of both estrogen receptors (ERs), α and β, producing two opposite but anticancer effects. Its effect ERα influences the PI3K/AKT/mTOR and RAS/ERK signal transduction pathways and thus induces autophagy, while it increases the numbers of apoptotic cells upon acting on ERβ [76]. Similarly, the phytochemical also inhibits metastasis via suppression of epithelial to mesenchymal transition (EMT) by suppressing TGF-β2 expression. Its anti-metastatic effect is also mediated via the Jak2/STAT3 pathway [76]. The potential therapeutic role of silibilin in disrupting metabolic homeostasis in triple-negative breast cancer cells by modulating the EGFR-MYC-TXNIP axis has also been reported [77].

The ability of thymoquinone, derived from Nigella sativa, to inhibit the bone metastasis of breast cancer cells was reported by Shanmugan et al. (2018) [78], The compound mediates this abrogation of the CXCR4 signaling axis as well as NF-kB [78].

Garcinol, a polyisoprenylated benzophenone, obtained from Garcinia indica has been shown to initiate apoptosis in MCF7, MDAMB231, and SKBR3A breast cancer cell lines by downregulating the expression of anti-apoptotic proteins such as Bcl-XL and Bax. Its mechanistic role in inducing cell cycle arrest and apoptosis in Her-2 over-expressing breast cancer cells has been reported by Aggarwal and colleagues (2020) [79]. The ability of the compound to cause loss of mitochondrial fragmentation and its transmembrane potential (ΔΨm) and apoptosis in MCF-7 cells has also been revealed [79].

Sulforaphane, a natural compound derived from cruciferous vegetables such as Brussels sprouts and broccoli known for its antioxidant and anti-inflammatory potentials was established, both in in vivo and in vitro investigations, to suppress the growth and proliferative ability of TNBC and BCSCs. Specifically, BCSC-associated pathways such as wnt/β-catenin and Notch pathways are disrupted by the compound [80].

Generally, various phytochemicals employ different mechanisms to suppress the growth and survival of breast cancer cells and specifically target such pathways as Wnt/β-catenin Hippo, Notch, and Hedgehog signaling pathways as well as JAK/STAT and PI3K/AktmTOR pathways, shutting down breast cancer stem cells to suppress the aggressiveness, heterogeneity, and remission of breast cancer cells.

## 6. Chlorogenic Acid and Cinnamaldehyde

**Figure 2:**
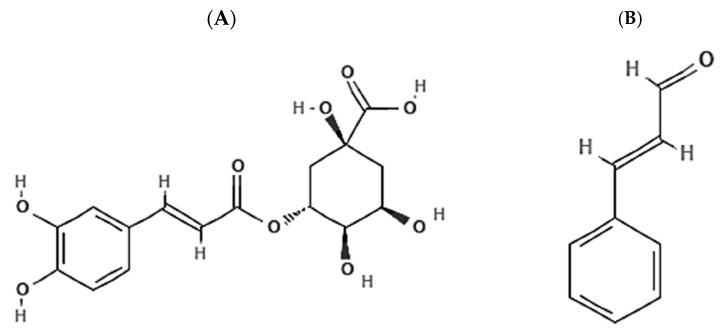
Chemical structures of (**A**) chlorogenic acid and (**B**) cinnamaldehyde.

The term “Chlorogenic acids” is used to describe a big class of polyphenolic compounds formed from the esterification reaction between trans-cinnamic acids such as ferulic acid, caffeic acid, and coumaric acid on one hand, and quinic acids (1-hydroxyhexahydrogallic acid) such as feruloyl quinic acid, caffeoylquinic acid, and coumaroylquinic, on the other hand. Chlorogenic acids in each subclass exist in different isomeric forms, giving rise to several molecular compounds in the class. However, the most abundant isomer in plant sources is 5-O-caffeoylquinic acid (5-CQA). Because of its abundance and availability, 5-CQA is currently called chlorogenic acid (CGA) (Figure 2A). CGA is an ester or depside acid produced as an intermediate in lignin biosynthesis in the plant shikimate pathway. CGA is sparingly soluble in non-polar solvents like ether, chloroform, and benzene but dissolves readily in aqueous solvents like ethanol, methanol, and acetone. The dietary sources of CGA include coffee beans, potato tubers, sweet potato leaves, eggplant, artichoke, sunflower seed kernels, cork, Eucommia leaves, chrysanthemum, strawberry, mango, blueberries, mulberry leaves, and so on. Several studies, in vitro and in vivo, have demonstrated the various biological activities of the compound. These include free radical-scavenging effects, anti-tumor, anti-bacterial, and anti-inflammatory effects, regulation of lipid and sugar metabolism, and protection of the nervous system [81,82,83,84,85].

On the other hand, cinnamaldehyde (also called trans-cinnamaldehyde and cinnamic aldehyde) is a bioactive agent occurring naturally in the bark of cinnamon trees and other plants of the genus Cinnamomum. Cinnamaldehyde (CA) (Figure 2B) is the chemical compound that gives cinnamon its characteristic flavor and odor. It is about 90% of the essential oil obtained from the bark of cinnamon trees. CA is an α, β-unsaturated aldehyde, a phenylpropanoid, biosynthesized by the shikimate pathway by the plant species in the genus Cinnamomum. It is a yellow oily viscous liquid, with a viscosity higher than water. It is hardly soluble in water but dissolves in organic solvents such as acetic acid, dimethyl sulfoxide, propylene glycol, and ethyl alcohol. CA has high volatility and becomes oxidized to cinnamic acid upon exposure to an oxygen-filled milieu—this explains its instability in the bloodstream. Studies have indicated that CA has a plethora of biological activities such as anti-inflammatory, antioxidant, and antitumor effects, regulation of blood lipid and glucose metabolism, improvement of cardiovascular diseases, autophagy, and apoptotic modulatory effects, and regulation of epigenetic reprogramming among others [17,86,87,88].

### 6.1. Chlorogenic Acid and Cinnamaldehyde as Effective Chemo-Preventive and Therapeutic Agents in Breast Cancer

Chlorogenic acid and cinnamaldehyde have been proven to have several molecular targets in breast cancer cells to inhibit the overall survivability of the mammary tumor. Noteworthy is the ability of these compounds to destroy tumor cells and shut down their ability to re-emerge. This is particularly important considering the fact that the majority of breast cancer treatment options are not able to effectively destroy breast cancer progenitor cells that later repopulate and constitute new malignant cells. Also important is the ease of procuring these phytochemicals, either commercially as pure compounds or from whole plants containing these bioactive agents. The compounds have been reported to target multiple cellular signaling pathways that are responsible for molecular events that characterize breast cancer cells.

The hallmarks of breast cancer include replicative immortality, resistance to cell death, evasion of growth-suppressing signals, sustained cell growth and division signals, induction of angiogenesis, activation of invasion and metastasis to secondary sites, deregulation of cellular energetics and general metabolism, evasion of the immune system, activation of tumor-promoting inflammation, genome instability, epigenetic reprogramming, the establishment of the tumor microenvironment, and deregulation of the endocrine system. Cancer is defined, generally, as a “collection of multiple diseases” since several disorders or “deviations from normal body homeostasis” co-exist at the same time within the same tissues. Effective treatment options will be ones that can target or combat the several “deviations from normal body homeostasis”. In other words, effective treatment options will be those that can target those identified cancer hallmarks. Current breast cancer treatment modalities can target one or a few of the hallmarks but leave other hallmarks intact at least, or to flourish at worst, thereby letting the latter promote breast cancer remission. This is a bane to effective breast cancer treatment [89,90,91,92,93,94].

Natural compounds such as CGA and CA have proven their efficacy in combating the known hallmark of breast cancer (Figure 1). They have proven to be effective as chemo-preventive agents. Interestingly, they can act as both blocking agents—preventing the initiation of tumorigenesis through such mechanisms as the detoxification of carcinogens, the prevention of DNA adduct formation, scavenging electrophilic species, the prevention of lipid peroxidation, and the protection of mutagenesis among others—and suppressing agents—repressing the promotion and development of preneoplastic tissues through mechanistic processes like the promotion of apoptosis and autophagy, inhibition of breast cancer cell migration and invasion, disruption of cancer energy metabolism, and blockage of the estrogen receptor [14,81,87,95]. CGA and CA therapeutic potentials have been reported in various in vivo and in vitro investigations. The phytochemicals can affect the promotion of apoptosis, cell cycle arrest, inhibition of invasion and metastasis, inhibition of vascularization, promotion of autophagy, inhibition of cell proliferation, regulation of the endocrine system, restoration of normal cellular metabolism, suppression of drug efflux proteins, inhibition of breast cancer stemness capability, and restoration of normal epigenetic markers among others in breast cancer cell lines and neoplastic tissue in experimental animals (Table 1).

One of the problems associated with breast cancer treatment is breast cancer invasiveness and metastasis. Metastasis is the villain in the disease’s poor prognosis [29,60]. Xue et al. [96] reported the ability of CGA to inhibit EMT, a molecular event that is necessary for epithelial cells to switch to mesenchymal cell-like migratory capacity, in breast cancer. CGA shuts down EMT via downregulation of low-density lipoprotein receptor-related protein 6 (LRP6), a component of the canonical Wnt/β-catenin pathway LRP6/LRP5/Frizzled receptor complex. The compound also reduces β-catenin expression as well as cell migration-promoting proteins N-cadherin and vimentin MMP-2 and 9, proteolytic enzymes used by invading cancer cells to degrade surrounding basement membrane structure [96]. Expression levels of E-cadherin and Zonula occludens-1 (ZO-1), junctional adaptor proteins that maintain adherence of epithelial cells to one another and thus render them non-motile, are upregulated by CGA [96,97]. Zheng et al. (2021) [98] also established the anti-metastatic potential of CGA in breast cancer via the downregulation of EMT [98].

Interestingly, one of the mechanisms through which cinnamaldehyde elicits its anticancer effect is EMT inhibition. CA can promote E-cadherin expression levels in tumor cells [118]. E-cadherin suppression is essential for the acquisition of the metastatic ability of breast cancer cells, according to Padmanaban et al. [99]. The anti–metastatic role of cinnamaldehyde via EMT suppression in various types of cancer has been previously reported in both in vivo and in vitro experiments [21,119,120].

CGA can disrupt cell cycle progress in cancer cells by upregulating tumor suppressor proteins such as p53 and p21 and downregulating the MAPK pathway [13,15,97,100]. Through the transactivation of its target genes involved in the induction of cell cycle arrest and/or apoptosis, activated p53 promotes cell cycle arrest to allow DNA repair and/or apoptosis to prevent the propagation of cells with serious DNA damage. The transcription factor is inducible by signals such as DNA damage and the activation of proto-oncogene [121,122]. p21 promotes cell cycle arrest by inhibiting the kinase activity of both CDK-1 and 2 as well as proliferating cell nuclear antigen (PCNA), required for the S-phase progression of the cell cycle. The protein can also trigger DNA damage repair before allowing the cell cycle to continue [122,123,124]. p53 and p21 are components of the p53-21-RB-E2F signaling network checkmating cell cycle progress. p53 and retinoblastoma protein (RB) are key tumor suppressors important in regulating the cell cycle. They are frequently mutated in breast cancer and other types of cancer. RB interacts with the E2F family of transcription factors to form complexes that can suppress the expression of genes promoting cell growth and division. Upon p53 activation, caused by DNA damage, for example, the expression of the p21 gene is upregulated, which then results in RB-E2F complex formation and subsequent downregulation of a set of genes driving cell cycle progression. Cell cycle arrest will allow for DNA damage repair or commit the cell to apoptosis if the damage is too much to be repaired [124]. The cell cycle-inhibitory capacity of CA has been reported in breast cancer and other types of cancer in previous investigations [19,101,102]. Neagle et al. [19] found that CA causes cell cycle arrest by inhibiting G2/M phase transition and spindle assembly. This occurs due to downregulation of CDK1, cell division cycle 25 (CDC25), CDC20, and survivin expression [19]. Jeong et al. [102] similarly reported that CA causes cell cycle arrest in G2/M progression [102].

CA and CGA promote breast cancer cell death via mitochondria-mediated apoptosis. The phytochemicals can inhibit the activities of anti-apoptotic proteins such as Bcl-2 and Bcl-xL while promoting the activity of pro-apoptotic proteins such as Bak, Bax, and Bid. The latter changes the outer membrane permeability transition pore to allow the release of cytochrome C from the inner mitochondrial membrane leaflet. Once released into the cytosol of the cell, a protein complex known as an apoptosome is formed and then activates caspase 9. Ultimately, effector caspase 3 is activated and the cancer cell is triggered to commit to apoptotic death irreversibly [125]. CA’s pro-apoptotic impact on TNBC cells and other types of cancer was reported previously [19,43,98,124]. Yi et al. [101] reported that CA promotes intrinsic apoptosis in TNBC and luminal subtype breast cancer via inhibition of the JAK2/STAT3/cMyc pathway [101]. Signal transducers and activators of transcription 3 (STAT3) is a transcription factor that upregulates the expression of the c-MYC oncogene, cell cycle driver protein cyclin D1, and the anti-apoptotic protein Bcl-2 gene [101,126]. Inhibition of the pathways allows apoptosis to be activated in breast cancer cells. Several investigations revealed the roles of CGA in promoting cell death pathways in different types of breast cancer subtypes and other types of cancer cells [11,16,43,81,98,104,127]. Zheng et al. [98] reported the anti-apoptotic impacts of CGA in a subcutaneous tumor mouse model of 4T1 cells [98]. CGA was able to induce apoptosis by downregulating the release of Bcl2 while upregulating the release of pro-apoptotic protein Bax with concomitant increased expression of p53 and caspase 3 in 4T1 breast cancer cells in BALB/c mice [103]. It is important to note that there is more information on mitochondrially induced apoptosis in colon cancer [128] and leukemias [129], perhaps because they are consumed. There is much less information specifically related to this mechanism of action from CGA and CA in breast cancer. While other studies included in this review discuss apoptosis and cell death, no clear delineation is made between extrinsic and intrinsic pathways. Therefore, more study is needed to elucidate mechanisms inducing cell death due to treatment with CGA and CA.

Solid tumors such as breast cancer require vascular systems, which include blood and lymphatic vessels, for their growth and metastasis. Vasculatures are important in nutrient obtainment, oxygenation, and waste removal by solid tumors. Angiogenesis is, therefore, a sine qua non for the overall survival of mammary tumors and other types of neoplastic tissues. Vascularization is a challenge to solid tumor treatment [130,131]. Phytochemicals such as CGA and CA shut down pathways promoting angiogenesis in breast cancer and other types of cancer cells [104,105,106,132]. Studies have revealed the ability of CGA to act as an anti-angiogenic agent by inhibiting vascular endothelial growth factor (VEGF) and VEGF receptor 2-mediated signaling pathways [105]. Via the downregulation of the Akt/HIF-1α pathway, CGA can prevent hypoxia-driven vascularization [104]. Lu et al. [132] found that cinnamaldehyde elicits an anti-angiogenic impact in cancer cells by shutting down VEGF-mediated signaling by blocking VEGFR [132]. The ability of CA to downregulate the expression of HIF-1α and thereby prevent vascularization in tumors has been reported [106]. With unregulated growth and division of cells in the tumor microenvironment (TME), the cell number and volume increase unceasingly; as a result, the cells in the TME experience hypoxic conditions. As hypoxia sets in, hypoxia-inducible factor-1α (HIF-1α) expression is stimulated via an Akt-dependent pathway, which in turn switches on the angiogenic program. This results in the activation of inflammatory signaling and the recruitment of pro-inflammatory cytokines and vascular cells to the TME and the resultant transcription of genes that are key to vascularization such as platelet-derived growth factor (PDGF), VEGF, and MMPs [130,131]. However, these phytochemicals can inhibit HIF-1α, PDGF, VEGF, and MMPs and thus inhibit angiogenesis in cancer cells [104,105,106,132].

Breast cancer stem cells, a small population of breast cancer cells with self-renewal and strong proliferating capabilities, are the drivers of breast cancer heterogeneity and remission. BCSCs constitute the biggest impediment facing conventional breast cancer treatment methods to achieve effectiveness. These subpopulations of cells are the clog in the wheel of holistic breast cancer treatment as they repopulate the tumor tissue, thereby causing cancer remission. Evidence has pointed to Notch, Wnt/β-catenin, JAK/STAT, and Hedgehog signaling pathways as the key pathways driving the stemness characteristics of BCSCs. Targeting these pathways holds the key to effectively destroying the BCSCs and in turn completely treating breast cancer [105,133,134]. CGA and CA are potential candidates capable of targeting signaling pathways governing the stemness of BCSCs as suggested by previous investigations [135,136,137].

Cancers, regardless of their cellular or tissue origin, are characterized by metabolic reprogramming, which promotes both tumor growth and invasion [107]. The metabolism of cancer cells is characterized by their ability to acquire nutrients from nutrient-poor environments for maintaining viability and clonal expansion. Alterations in intracellular and extracellular metabolites associated with cancer-associated metabolic reprogramming may profoundly influence gene expression, cellular differentiation, and the tumor microenvironment. In addition to their metabolic plasticity, cancer cells and their development into full-blown malignancy and metastases are characterized by their context-dependent diversity of phenotype. According to Lasche et al. [138] and Pavlova and Thompson [139], several characteristic features of cancer metabolism can be identified such as (1) increased glucose and amino acid uptake and metabolism to fulfill their energy need and their requirement for nitrogenous compounds, (2) glycolysis and TCA cycle reactions are increased to generate metabolic intermediates that are needed to build biomolecules essential to support their increased rate of growth and division, (3) acquisition of alternative means of obtaining nutrients to meet their aggressive nutritional needs, and (4) reprogramming of cancer cell metabolism by tumor-driven growth and survival signals to increase nutrient acquisition and biosynthesis [138,139]. Phytochemicals have been reported to modulate re-wired cancer metabolism. They exert effective anticancer activity by affecting key glycolytic pathway regulators, including hexokinases, glucose transporters, lactate dehydrogenase, pyruvate kinase, and phosphofructokinase. Studies have also indicated that phytochemicals can mediate HIF-1α expression and metabolic activities as well as PI3K/Akt/mTOR and MAPK pathways associated with HIF-1α [107]. Kim et al. (2020) [108] reported the role of CA in modulating cancer metabolism through the inhibition of hexokinase and pyruvate kinase and STAT3 signaling [108]. In a similar finding, Schuster et al. [43] revealed that both CGA and CA destroy TNBC and luminal-subtype breast cancer cell bioenergetics and suppress their overall survival [43].

### 6.2. Multidrug Resistance in Breast Cancer Cells: The Potential Intervention of Chlorogenic Acid and Cinnamaldehyde

Multidrug resistance (MDR) is described as the main mechanism by which cancer cells develop resistance to therapeutic agents. It is a major factor hindering the effectiveness of many types of breast cancer treatment modalities. Chemotherapy, one of the conventional breast cancer treatment options, has been greeted with failure in many cases of breast cancer treatment owing to the latter’s development of resistance to the drug, thus resulting in malignant aggressiveness of the neoplastic tissue. MDR has been observed in both solid tumors such as breast and ovarian cancers, and hematologic cancers such as lymphoma and leukemia. Because of the intra-tumor heterogeneity of breast cancer cells, some breast cancer cell populations may be susceptible to drug cytotoxicity, while others may develop resistance. Thus, while drug-sensitive populations are killed, the drug-resistant subpopulation will survive and overpopulate the tumor, thereby resulting in full-blown drug-resistant breast cancer tissue [140,141].

One of the complications associated with breast cancer is the unavailability of breast cancer chemo-preventive or therapeutic measures in some countries of the global south, while patients in advanced countries can secure breast cancer treatments, they are faced with the problem of increasing breast cancer chemotherapy resistance development. MDR is a big challenge in breast cancer treatment delivery and research, in a manner likable to antibiotic resistance development. Several mechanisms are employed by cancer cells to mount resistance against therapeutic agents. Identified mechanisms of MDR include increased drug efflux, reduced cellular drug uptake, altering drug metabolic pathways, increased drug detoxification, modification of drug targets, drug sequestration, amplification of target genes, and epigenetic reprogramming, among others [140,141,142,143].

One of the major mechanisms of MDR is the efflux of anticancer drugs by a family of transmembrane transporters known as ATP-binding cassette (ABC) transporters. They are translocases using ATP binding and hydrolysis to transport drugs across the cell membrane. They are transport systems in humans for pumping out drugs from the intracellular milieu. There are dozens of ABC proteins in humans, with the widely known members being P-Glycoprotein (P-gp), breast cancer resistance protein 1 (BCRP1), and multidrug resistance protein 1 (MRP1) [144,145]. Studies have revealed that these transporters are overexpressed in many cancer cells such as breast cancer cells. Their overexpression has been described as the main cause of MDR in cancer cells. P-gp, MRP1, and BCRP1 are widely used by breast cancer cells to pump anticancer agents out of the breast cancer cells and are targets of breast cancer therapeutic research. Inhibiting the expression and/or activities of these translocases is imperative for the development of effective breast cancer treatment strategies [141,145,146].

Several studies have indicated the role of phytochemicals in increasing the sensitivity of the cancer cell population and overcoming their resistance to drug agents. Various plant-based bioactive agents have demonstrated abilities to suppress MDR in breast and other types of cancer cells by downregulating the expression and/or activity of P-gp, MRP1, and BCRP1 transporters. Phytochemicals can be used, alone or in synergism with other phytochemicals or as adjuvants with known anticancer drugs with known molecular targets, to kill breast cancer cells [145,146,147].

The ability of chlorogenic acid and cinnamaldehyde to suppress drug resistance development in cancer cells and beyond has been indicated by previous investigations [12,109,148,149,150,151,152]. Toumia et al. [153] reported on the ability of CGA-containing plant extract, as an adjuvant, to effectively suppress MDR in cancer cells by downregulating the expression of P-glycoprotein [153]. Ciudad-Mulero et al. [100] indicated that the PI3K/AKT/mTOR/PTEN signaling axis induces the expression of multidrug resistance-associated protein and that CGA could modulate the activity of the PI3K/AKT/mTOR/PTEN pathway [100]. Several studies have reported the ability of CGA to regulate Akt-mediated signaling pathways in breast cancer cells [98,104,154,155,156]. CA similarly has modulatory effects on PI3K/AKT/mTOR signaling networks in other cancer types [18,21,157,158,159]. Thus, by extension, it is predicted to have the same ability to suppress MDR in breast cancer cells. Via downregulation of Akt and STAT3 pathways, CA derivate was reported to suppress the expression of P-gp in triple-positive drug-resistant breast cancer cells [110,111]. The compound, as an adjuvant, downregulates the expression of the ABC transporter and resultantly promotes the uptake of an anticancer agent, which then results in reduced levels of anti-apoptotic proteins such as survivin, Bcl-2, and Bcl-xL and increased levels of activated caspase 9, thus promoting the breast cancer cells’ apoptotic death [110,111].

### 6.3. The Protective Abilities of Chlorogenic Acid and Cinnamaldehyde in Epigenetic Reprogramming-Driven Breast Cancer Cells

Epigenetics is defined as heritable alterations in gene expression without changes to the DNA sequence. These include changes in DNA methylation, histone modification, miRNA expression, and chromatin remodeling. Epigenetic changes have been implicated in the development and progression of different types of cancers. Active investigations in the field of epigenetics have expanded our understanding of how epigenetic reprogramming affects patterns of gene expression and the development of diseases such as breast cancer [160]. Alterations in epigenetic markers in the genome such as CpG island methylation and histone modification, importantly, appeared to be conserved in the genome and transmittable through cell cycles to the daughter cells. Studies have revealed that several epigenetic modifications in tumor suppressor genes such as p53 and BRCA 1/2 contribute significantly to the cancer epigenome in cancer patients and/or their descendants after several years [161]. Alteration in the epigenome can be manifested through the suppression of tumor suppressor genes and/or oncogenes and their consequent phenotypic impact on cellular activities. Several biomolecular processes could be disrupted because of epigenetic reprogramming. Epigenetics plays a significant role in cancer biology [162]. Studies have shown the roles of alteration in epigenetic mechanisms in the over-expression and over-activation of endocrine receptors in breast cancer cells as well as the EMT and the acquisition of migratory ability. The impact of epigenetic changes in genes involved in DNA damage repair and angiogenesis such as HIF-1α and VEGF; apoptotic genes such as Bak and Bcl-xL; and genes involved in the production of xenobiotic-metabolizing enzymes have been established [160,161,162,163].

The epigenetic landscape is wide and offers vast opportunities for the development of cancer therapy. The roles of various natural compounds in modulating the epigenetic signature in breast cancer and other types of cancer have been reported. The modulatory effects proceed by regulating the enzymes responsible for DNA and histone modifications such as DNA methyltransferases (DNMTs) and histone methyltransferases (HMTs) and mechanisms of chromatin remodeling. The anticancer activities of bioactive agents can be manifested by the reversal of altered epigenetic markers associated with the inactivation of tumor suppressor genes and activation of proto-oncogenes [164,165].

Hernandes et al. [166] revealed that CGA restored the CpG hypomethylation status of cancer and then predisposed the cancer cells to its cytotoxic and genotoxic effects [166]. Ding et al. [167] asserted that CGA modulates gene expression patterns by restoring normal epigenetic signatures such as CpG island DNA methylation, histone post-translational modification, and microRNA expression [167]. In their investigation of the roles of CGA on epigenetic reprogramming in breast cancer, Lee and Zhu [112] found that CGA inhibits DNA methyltransferase (DNMT) to prevent hypermethylation of the DNA sequence in both triple negative and triple positive breast cancer cells [112].

Bartolomeu et al. [113] reported that CGA, in combination with caffeine, attenuates carcinogenesis via the downregulation of a known oncogenic microRNA (oncomiR), miR-21a-5p. Downregulation of the epigenetic marker manifested in reduced cell proliferation and upregulation of apoptosis in the tumor as well as suppression of inflammation. The group revealed that the cognate mRNA target of miR-21a-5p has a tumor suppressor function [113]. The ability of CGA-containing coffee to shut down the cancer-promoting MAPK pathway was demonstrated by Nakayama et al. [114]. The effect was elicited by promoting the expression of two microRNAs, miR-30c and miR-96, both of which target the KRAS proto-oncogene, a component of the MAPK signaling network [114].

The role of CA in the modulation of epigenetics in cancer cells has been reported in previous investigations. Wang et al. [115] demonstrated that CA can suppress the invasive capabilities of TNBC through the downregulation of tumor-promoting microRNA, miR-27a, expression [115]. Similarly, Chen et al. [116] reported the ability of CA to inhibit cancer cell proliferation, migration, and invasion and promote apoptosis. The anticancer effect was indicated to proceed through the modulation of epigenetic markers. The phytochemical inhibits the expression of several oncogenic microRNAs and long non-coding RNAs as well as upregulation of certain tumor-suppressor microRNAs and long non-coding RNAs [116]. An interesting finding was made by Tian et al. [117] when they reported that CA was able to induce the expression of circular RNA, circRNA, to elicit its antitumor effect. They reported that a novel endogenous circular RNA, hsa_circ_0043256, was involved in promoting apoptosis and inhibiting the Wnt/β-catenin pathway in cancer cells. The circRNA was revealed to function as a miRNA sponge to block miR-1252. The miR-1252 targets ITCH mRNA for gene silencing. This suggests that CA inhibits the activity of miR-1252 by promoting the expression of the circRNA. Thus, CA asserts its anticancer effect in the cancer cells via the hsa_circ_0043256/miR-1252/ITCH epigenetic network [117].

### 6.4. Synergism of CGA and CA in Breast Cancer

Based on xenobiotic pharmacodynamics, the interaction between two (or even more) phytochemical agents, just like drugs, could be positive, negative, or even neutral. Positive interaction often termed potentiation is the enhancement of the biological effects of one phytochemical agent by another to bring about increased net pharmacological impacts of the two phytochemicals. Potentiation could be additive or synergistic. Additivity is when the interaction of two phytochemicals produces a combinatorial effect that is the sum of the biological effects of each of the phytochemical agents. Synergism, on the other hand, is an interaction that results in each phytochemical producing biological effects that are bigger than the sum of the individual biological activities. Negative interaction is an antagonistic interaction that produces a net effect that is smaller than the biological effects of each of the components. Inhibition of the full biological impact of a compound by another is antagonism [168,169].

Many phytochemicals found in different plants may have evolutionary significance due to their complex interactions with one another, which may work synergistically to mechanistically provide chemo-preventive and therapeutic effects against diseases such as breast cancer [169,170,171]. Synergistic interaction is often seen when phytochemicals are combined in disease prevention and treatment. Synergism is indicative of the bioactive agents employing different mechanisms of action to elicit their effect or interact with different molecular targets [170,171]. Whole plants, such as fruits and vegetables which contain a mixture of several phytochemicals, can demonstrate higher biological effects compared to individual isolated or purified phytochemical agents because of synergistic interaction between the plethora of natural compounds they contain [4].

A combination of different phytochemical agents with known anticancer capabilities will produce more effectiveness in their cancer-killing activities because of their synergistic interaction. This phytochemical synergism in destroying cancer cells has been reported by many investigators. Evidence from omics and molecular studies revealed that phytochemical mixtures produce combinatorial effects by targeting multiple molecular targets in cancer cells [170]. Tian et al. (2010) [172] reported that a combination of several plant-natural compounds shut down differentially expressed proteins in the MCF-7 human breast cancer cell line and promoted apoptotic cell death [172]. Similar findings were reported by Zhang and colleagues (2004) [173] that phytochemical mixture downregulates the expression of dozens of tumor-promoting genes and shuts down MAPK, PDGF, and Notch pathways among others and significantly promotes apoptosis in cancer cells [173].

Through synergistic interaction with hesperidin, CGA promotes breast cancer death by modulating the estrogen receptor/mitochondrial pathway [16]. The CGA and piperine mixture stops the proliferative activity of carcinoma via mitotic inhibition, the promotion of cell cycle arrest, and the downregulation of several genes promoting cancer cell invasiveness [174]. CA synergistically interacts with berberine to elicit a chemo-preventive role by shutting down tumor bioenergetics by upregulating AMP-dependent protein kinase (AMPK) and mTOR pathways while inhibiting the NF-κB signaling axis [175]. Milani et al. [176] established that CA and epigallocatechin impose cytotoxicity on cancer cells in a synergistic manner [176].

Synergism of both chlorogenic acid and cinnamaldehyde was established in different subtypes of breast cancer in in vitro investigations by Schuster et al. [43]. CGA and CA were reported to destroy energy metabolism and survivability in different breast cancer subtypes by altering mitochondria membrane potential difference, reducing glucose metabolism and ATP generation, increasing free radical generation in mitochondria, and changing the overall cancer cell morphology, thus resulting in apoptosis [43]. Furthermore, the two compounds synergistically inhibit the invasive and migratory capacity of both TPBC and TMBC, MCF7, and MDA-MB-231 cell lines. Western blotting analysis of protein expression indicated that the switch from an epithelial cell to a mesenchymal cell characteristic was induced by the compounds in a manner that is mediated by inhibition of Akt (protein kinase B) activation, as evidenced by decreased phosphorylated Akt expression in both cell lines. As highlighted previously, the Akt pathway is central to the malignancy of various breast cancer cells, and thus, the inhibition of the pathway, as seen in the case of the CGA and CA combination, is an effective target to arrest the malignancy and metastasis of breast cancer cells. Similarly, growth curve analysis indicated that the compounds inhibited the proliferative capacity of the cells. Fluorescence microscopy of the annexin V- and propidium iodide-stained cells further indicated the ability of the compounds, combinatorically, to cause cytotoxicity to the cancer cell lines (unpublished data [177]).

## 7. Conclusions and Future Directions

Chlorogenic acid and cinnamaldehyde demonstrate effectiveness in affecting multiple signaling pathways and molecular targets in delivering cytotoxicity to all breast cancer subtypes. They exert their anticancer effects through the promotion of cell death and autophagy, inhibition of cell proliferation, suppression of invasion and metastasis, induction of cell cycle arrest, prevention of angiogenesis, restoration of normal cellular energetics and metabolism, inhibition of multidrug resistance development, modulation of epigenetic mechanisms, and killing of cancer stem cells in breast cancer cells. Importantly, their cell-killing activities proceed without affecting normal cells and by rendering the breast cancer cell unable to recur.

**Figure 3:**
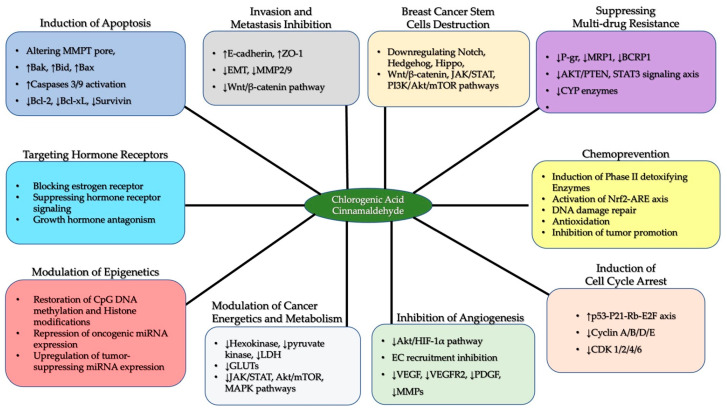
Mechanisms of actions of chlorogenic acid and cinnamaldehyde in breast cancer cells. Chlorogenic acid and cinnamaldehyde assert their breast cancer-killing capacity via various mechanistic processes. As indicated above, the compounds impact several molecular targets and pathways in breast cancer cells to shut down their overall survival. Up arrow indicates upregulation. Down arrow indicates downregulation.

The synergism of the two small molecules raises their biological activities and reinforces their capacity to target the vulnerabilities of breast cancer cells. Phytochemicals such as CGA and CA, especially when combined, are therefore presented as the much-needed “Holy Grail” that will work magically like the German Paul Ehrlich’s “magic bullet” that will not only optimally kill cancer cells but also prevent cancer relapse, while sparing normal cells from their cytotoxicity. These activities are summarized in Figure 3.

As effective as the anticancer potential of these compounds is, most of the previous investigations on these bioactive agents were conducted in vitro. While the in vitro research model system is highly advantageous in establishing proof-of-concept and pilot data, it does not reflect the inherent complexity of a physiologically relevant model. Thus, it becomes pertinent to examine the cytotoxicity of the compounds against breast cancer cells in vivo. Conducting in vivo studies is imperative in holistically understanding the pharmacokinetics and pharmacodynamics of xenobiotics in translational biomedical research. Therefore, the next logical step is to investigate the anticancer capabilities of chlorogenic acid and cinnamaldehyde synergism in an experimental model such as a mouse model to continue moving forward from bench to bedside.

Such future studies will seek to also investigate the pharmacokinetics of the compound. The studies will examine the effect of gastrointestinal pH on the stability of the compounds, the impact of the hepatic first-pass metabolism on the compounds, and thus the bioavailability of the compounds in the systemic circulation. Similarly, such a future investigation will examine if there is a need to use delivery vehicles, such as nano-transport systems, for targeted delivery of the compounds to enhance maximum delivery of the compounds to the breast cancer cells and allow for optimum efficacy of the anticancer agents.

## Figures and Tables

**Table 1:** Mechanisms of action of chlorogenic acid and cinnamaldehyde in breast cancer cells.

Anticancer Effect on Breast Cancer Cells		Mechanisms of Action			
		Chlorogenic Acid	Ref	Cinnamaldehyde	Ref
1.	Anti-invasion and anti- metastasis	1. Inhibition of EMT 2. Downregulation of LRP6, the part of canonical wnt/β-catenin pathway receptor complex	[96,97,98]	Promotion of E-cadherin expression	[99]
2.	Halting cell cycle progress	1. Upregulation of the expression and activity of tumor suppressor proteins p53 and p21 2. Downregulation of the MAPK pathway	[13,15,21,97,100]	Inhibition of CDK1, CDC25, CDC20 and survivin expression resulting in: 1. Arrest at G2/M transition 2. Disruption of spindle assembly formation	[19,101,102]
3.	Promoting apoptosis	Promoting mitochondrial-mediated cell death via: 1. Downregulation of Bcl-2 and upregulation of Bax 2. Stabilization of p53 and caspase 3 activity	[98,103]	Driving the intrinsic apoptotic pathway via: The downregulation of Bcl-2 through the downregulation of the JAK2/STAT3/cMyc pathway	[101]
4.	Anti- angiogenesis	1. Blockage of the Akt/HIF-1α pathway 2. Downregulation of VEGF and VEGF receptor-2 mediated signaling.	[104,105]	Preventing the stabilization of HIF-1α	[106]
5.	Modulation of rewired cancer metabolism	Regulation of the PI3K/Akt/mTOR pathway and HIF-1α expression	[43,107]	1. Inhibition of the activities of hexokinase and pyruvate kinase, key glycolytic enzymes. 2. Suppression of STAT3 signaling	[108]
6.	Suppression of multi-drug resistance	1. Downregulation of the expression of P-glycoprotein 2.Modulation of the PI3K/AKT/mTOR/PTEN signaling axis to shut down ABC transporter expression	[100,109]	Suppression of the P-gp ABC pump via the regulation of Akt/STAT3 signal transduction	[110,111]
7.	Regulation of epigenetic programming	1. Prevention of DNA hypermethylation via inhibition of the DNMT enzyme 2. Downregulation of oncogenic microRNA (oncomiR) such as miR-21a-5p. 3. Promotion of the expression of tumor-suppressing microRNAs (such as miR-30c and miR-96) to target oncogenic transcripts such as KRAS	[112,113,114]	1. Downregulation of onco-miR such as miR-27a 2. Promotion of tumor-suppressing microRNA and long non-coding RNA to target oncogenes 3. Induction of endogenous circular RNA expression to promote apoptosis and inhibit the wnt/β-catenin pathway.	[115,116,117]

## Data Availability

Data sharing is not applicable.
